# Multifaceted Insights into Innovative Approaches to Treating Colorectal Cancer Metastasis: From Emerging Biological Factors to Radiomics

**DOI:** 10.3390/cancers15184644

**Published:** 2023-09-20

**Authors:** Alessandro Ottaiano, Luisa Circelli, Mariachiara Santorsola, Michele Caraglia

**Affiliations:** 1 Istituto Nazionale Tumori di Napoli, IRCCS “G. Pascale”, Via M. Semmola, 80131 Naples, Italy; mariachiara.santorsola@istitutotumori.na.it; 2 AMES, Centro Polidiagnostico Strumentale srl, Via Padre Carmine Fico 24, 80013 Casalnuovo Di Napoli, Italy; genetica@centroames.it; 3 Department of Precision Medicine, University of Campania “L. Vanvitelli”, Via L. De Crecchio 7, 80138 Naples, Italy; michele.caraglia@unicampania.it

We extend our appreciation to the authors who have made substantial contributions to the Special Issue focusing on “Colorectal Cancer Metastasis”. Their work has furnished invaluable insights and presented intriguing data pertaining to the intricate phenomenon of colorectal cancer metastasis. Metastasis remains a pivotal determinant of unfavorable prognosis with respect to solid tumors; therefore, comprehending and dissecting the multifaceted factors that underlie the promotion of metastasis hold paramount significance in the development of innovative therapeutic strategies within the realm of oncology.

Di Grazia A. et al. [1] directed their attention towards hepcidin, a peptide hormone primarily synthesized in the liver by hepatocytes, although it is also produced by macrophages and enterocytes. Hepcidin mainly controls the absorption of dietary iron in the intestines and the release of iron from storage in the liver and spleen. Their study delves into the role of this peptide in driving processes associated with the biological and clinical aggressiveness of colon cancer. Notably, their study reports heightened levels of hepcidin in cases of metastatic colorectal cancer (mCRC). The survival analysis conducted in their study demonstrates a significant association between elevated hepcidin expression and a less-favorable prognosis. Furthermore, their research elucidates a correlation between hepcidin expression and markers indicative of epithelial-to-mesenchymal transition (EMT). Notably, the silencing of hepcidin in CRC cells induces a reduction in EMT markers, providing further insights into the mechanistic aspects of CRC metastasis. In recent years, there has been a growing focus on investigating the tumor microenvironment (TME), which comprises over 80% of a tumor mass’s cellular components and encompasses a variety of cell types, including lymphocytes, endothelial cells, macrophages, fibroblasts, and others. This work by Di Grazia A. et al. also emphasizes the intricate interplay within the TME, particularly the interaction between specific cytokines (such as IL-6, -10, -21, and -23) and hepcidin. Additionally, it underscores hepcidin’s role in fostering the accumulation of M2-polarized tumor-associated macrophages (TAMs), which are identified by the characteristic marker CD206. This has significant implications because the presence of M2 TAMs has been associated with a poorer prognosis in various cancer types and resistance to immunotherapies. Hepcidin is emerging as a compelling biomarker in the context of mCRC, with potential future applications in tailored therapeutic strategies.

The realm of biomarkers remains a continually evolving and profoundly captivating domain within oncology. Biomarkers have the potential to differentiate subgroups of metastatic patients at a higher risk of disease progression and mortality while also guiding the development of personalized therapeutic approaches. In this context, the research conducted by Lee S. et al. [2] stands out as particularly intriguing. Their study delved into the field of RNA sequencing within plasma exosomes derived from patients who had undergone liver metastasectomy for mCRC. The primary goal of this investigation was to unearth novel biomarkers that could shed light on disease progression in this complex clinical scenario. Exosomes, a subset of extracellular vesicles released by cancer cells, play pivotal roles in the intricate landscape of cancer progression and resistance to therapy. The authors of this study successfully demonstrated that exosomal CXCL10, originating from cancer cells, holds promise as a biomarker for liver metastasis caused by CRC. Moreover, it is emerging as a potential target for both prevention and treatment strategies. CXCL10, a member of the CXC chemokine family, exerts its biological effects by binding to the CXCR3 receptor. Alongside its counterparts in the CXC chemokine family, CXCL10 engages with G-protein-coupled receptors, initiating a wide spectrum of biological and physiological activities. The chemokine/receptor system is a highly captivating domain of exploration as it operates within both tumor cells and the TME. CXCL10 is intricately involved in processes such as chemotaxis, apoptosis induction, the regulation of cell growth, angiostatic effects, and the mediation of metastasis. Furthermore, multiple studies have linked it to drug resistance. Notably, akin to M2 macrophages, the signaling pathway of CXCL10 may establish an immunosuppressive niche, affording cancer cells the ability to proliferate while evading immune system attacks.

The research conducted by Descarpentrie J et al. [3] presents a captivating exploration of Proprotein Convertases (PCs) and their influence on the malignant behavior of colon cancer cells. PCs are pivotal enzymes responsible for converting inactive precursor proteins into their active forms, serving vital roles in numerous cellular processes. In the context of colon cancer, Descarpentrie and colleagues have unveiled notable alterations in the expression patterns of PCs within colon cancer stem cells (CSCs). Inhibiting the activity of these PCs has demonstrated a remarkable reduction in the growth, viability, and invasiveness of colon CSCs both in controlled laboratory settings and in live organisms. Notably, the suppression of PCs has also resulted in a significant reduction in the levels of stem cell markers, such as LGR5 and NANOG, within tumor tissues. Furthermore, the authors conducted an in-depth examination of furin, a specific PC, within colon cancer cases featuring *KRAS* or *BRAF* mutations. These clinical scenarios are of particular significance due to their heightened aggressiveness and resistance to conventional therapeutic approaches. In light of their findings, the authors propose that targeting furin may hold promise as a therapeutic strategy for managing colon tumors characterized by *KRAS* or *BRAF* mutations.

We express our gratitude for the valuable contribution made by Li J et al. [4]. Their work significantly contributes to raising awareness of the biological distinction between tumor mutational burden (TMB) and microsatellite instability (MSI). The authors have concentrated their efforts on early-stage CRC that has invaded lymph nodes (Stage IIIA), a category that has been notably underrepresented in extensive genomic mapping initiatives. Employing a comprehensive, massively parallel sequencing approach encompassing 409 key cancer-related genes, the authors demonstrate that high-burden microsatellite-stable (MSS) tumors exhibit co-occurring gene mutations (*ATM*, *ATR*, *CDK12*, and *PTEN*) that are not observed in low-mutational-burden microsatellite-stable cancers. Given the significance of ATM in homologous recombination deficiency (HRD), this study raises intriguing questions regarding the potential leverage of HRD in hyper-mutant MSS mCRC. These observations broaden our perspectives on the intricate genetic landscape of CRC and may pave the way for novel therapeutic approaches.

Granata et al. [5] present a study that investigates the utility of radiomic features derived from EOB-MRI phases (the delayed, hepatobiliary phase of magnetic resonance imaging) in predicting clinical outcomes subsequent to liver metastasectomies. This scientific exploration unfolds within a clinical and biological milieu of significant intrigue given the well-established knowledge that the surgical resection of liver lesions, following thorough multidisciplinary deliberations, can substantially augment patient survival and, in specific instances, offer a curative pathway in colorectal cancer management. Radiomics, a swiftly evolving domain, is committed to quantifying various metrics (radiomic features) from medical images. These radiomic features encapsulate essential tissue and lesion attributes, encompassing factors like heterogeneity and morphology. They can be harnessed, either in isolation or in synergy with demographic, histologic, genomic, or proteomic information, to address intricate clinical challenges. When delving into the study without delving into its intricate technicalities, it becomes evident that the authors have identified k-nearest neighbors (KNN), a particular pattern recognition technique, as the optimal approach for predicting clinical outcomes post liver metastasectomy.

In the near future, the amalgamation of our understanding of tumor biology, encompassing biochemical factors such as hepcidin, CXCL10, PCs, and others, with the burgeoning field of radiomics has the potential to yield robust predictive tools with profound implications for the field of oncology.

Immunotherapy serves as a potential catalyst that could redefine the landscape of oncological treatments, extending its influence even to mCRC. The conventional therapeutic paradigm, which previously limited its efficacy to patients with MSI, may soon find itself eclipsed. This shift is propelled by an increasingly profound comprehension of the intricacies of immunoresistance and immune editing within the intricate milieu of the TME. The work conducted by Garcia-Vicién G. et al. [6] plunges into the spatial intricacies of lymphocytic infiltrates within liver metastases, contextualized by the intricate tapestry of histologic growth patterns (HGP). Employing cutting-edge multispectral digital pathology techniques, this study meticulously characterizes three distinct localizations within the tumor landscape: the tumor periphery, the invasive margin, and the central tumoral areas. Within this landscape, the authors methodically dissect various histological sub-categories, including the central regions typified by mature stroma with low tumor cellularity, malignant cells interfacing with hepatocytes, regions exhibiting reactive stroma, fibrous capsule growth patterns, normal liver parenchyma, and diminutive tumor portions measuring 100 μm. Notably, the areas involving malignant cells in contact with hepatocytes (referred to as ndHGP) are predominantly populated by CD4+ cell subsets. This intriguing observation led to the hypothesis that these CD4+ cell subsets may possess immune-suppressive capabilities, opening up the possibility of their potential utility in immune-checkpoint-based therapy. While it is important to acknowledge that this study primarily serves as an exploratory endeavor, sparking hypotheses rather than drawing definitive conclusions, it undeniably lays the foundation for future investigations aiming to unravel the nuanced functional attributes of these distinct immune-cell subsets.

Finally, we express our appreciation to Seely KD et al. [7] for their valuable contribution to this Special Issue, shedding light on one of the most intriguing frontiers in oncological research: the intricate relationship between the gut microbiome and phenomena associated with tumor progression. Mounting evidence suggests that the diversity of bacteria does not merely promote carcinogenesis in primary colorectal cancer; it also exerts an influence on the advancement of metastases and the selection of target organs by reshaping the TME at both primary tumor sites and distant metastatic locations. The authors present a meticulously crafted and scientifically rigorous and critical review, offering readers a comprehensive understanding of the complex interplay between bacteria and the metastatic process. They have conducted a thorough examination of how bacterial infection influences carcinogenesis, tumor expansion, and the dissemination of cancer cells. Furthermore, their review of the literature has elucidated the intricate role of the lymphatic and venous systems in facilitating tumor metastasis under the influence of microbial factors. Notably, certain bacteria have demonstrated a remarkable capacity to enhance the EMT, a pivotal step in the metastatic cascade. Beyond this, bacteria can also actively shape the microenvironment and modulate the local immune profile at metastatic sites. Processes such as biofilm formation and coordinated migration along with quorum-sensing mechanisms enable bacteria to self-regulate, attract host cells, and establish a conducive, pre-metastatic niche at distant locations, thus fostering an environment conducive to tumor cell survival and proliferation. In light of these insights, the authors aptly propose that exploring early interventions, including targeted antibiotic therapy, holds promise as a strategy for mitigating metastatic dissemination in the presence of bacterial infections.

This Special Issue demonstrates how the phenomenology of cancer and metastasis is exceedingly complex, involving multiple scientific dimensions that, both from a prognostic and therapeutic perspective, are destined to be integrated in the future to enhance diagnostic and therapeutic strategies.

## References

[1] Di Grazia A., Di Fusco D., Franzè E., Colella M., Strimpakos G., Salvatori S., Formica V., Laudisi F., Maresca C., Colantoni A. (2022). Hepcidin Upregulation in Colorectal Cancer Associates with Accumulation of Regulatory Macrophages and Epithelial–Mesenchymal Transition and Correlates with Progression of the Disease. Cancers.

[2] Lee S., Park Y.S., Kim J.H., Lim A.R., Hyun M.H., Kim B., Lee J.W., Lee S.B., Kim Y.H. (2022). Identification of Biomarkers Associated with Liver Metastasis Progression from Colorectal Cancer Using Exosomal RNA Profiling. Cancers.

[3] Descarpentrie J., Araúzo-Bravo M.J., He Z., François A., González Á., Garcia-Gallastegi P., Badiola I., Evrard S., Pernot S., Creemers J.W.M. (2022). Role of Furin in Colon Cancer Stem Cells Malignant Phenotype and Expression of LGR5 and NANOG in KRAS and BRAF-Mutated Colon Tumors. Cancers.

[4] Li J., Steffen P., Tse B.C.Y., Ahadi M.S., Gill A.J., Engel A.F., Molloy M.P. (2022). Deep Sequencing of Early T Stage Colorectal Cancers Reveals Disruption of Homologous Recombination Repair in Microsatellite Stable Tumours with High Mutational Burdens. Cancers.

[5] Granata V., Fusco R., De Muzio F., Cutolo C., Setola S.V., Dell’Aversana F., Ottaiano A., Nasti G., Grassi R., Pilone V. (2022). EOB-MR Based Radiomics Analysis to Assess Clinical Outcomes following Liver Resection in Colorectal Liver Metastases. Cancers.

[6] Garcia-Vicién G., Mezheyeuski A., Micke P., Ruiz N., Ruffinelli J.C., Mils K., Bañuls M., Molina N., Losa F., Lladó L. (2022). Spatial Immunology in Liver Metastases from Colorectal Carcinoma according to the Histologic Growth Pattern. Cancers.

[7] Seely K.D., Morgan A.D., Hagenstein L.D., Florey G.M., Small J.M. (2022). Bacterial Involvement in Progression and Metastasis of Colorectal Neoplasia. Cancers.

